# Revenue-sensitive evaluation of AI-assisted ICD-10-CM coding and human-AI collaboration under dual DRG payment systems

**DOI:** 10.1038/s41598-026-57682-0

**Published:** 2026-06-11

**Authors:** Ming-I. Chen, Ying-Lin Hsu, Yi-Hsin Chen

**Affiliations:** 1 Doctoral Program in Big Data Analytics for Industrial Applications, National Chung Hsing University, Taichung, Taiwan; 2 Department of Applied Mathematics and Institute of Statistics, National Chung Hsing University, 145 Xingda Rd., South Dist., Taichung City, 402202 Taiwan; 3 Department of Nephrology, Taichung Tzu Chi Hospital, #88, Sec.1, Fengxing Road, Tanzi District, Taichung, 427215 Taiwan; 4 School of Medicine, Tzu Chi University, Hualien, Taiwan

**Keywords:** ICD-10-CM, Automated medical coding, Diagnosis-related groups, Revenue sensitivity, Human-AI collaboration, Natural language processing, Computational biology and bioinformatics, Diseases, Health care, Mathematics and computing, Medical research

## Abstract

Automated ICD-10-CM coding is critical for hospital reimbursement under Diagnosis-Related Group (DRG) payment systems, yet standard metrics weight all errors equally. This study evaluated 11 models on MIMIC-IV under heterogeneous conditions (the full 7942-code space, top-50 self-trained baselines, and 200-admission zero-shot LLM samples) and proposed two revenue-sensitive metrics: the Revenue Sensitivity Index (RSI) and Coding Reimbursement Score (CRS). Performance was compared across US Medicare Severity DRG (MS-DRG) and Taiwan DRG (Tw-DRG) systems, with five human-AI review strategies simulated. PLM-ICD achieved the highest micro-averaged F1 (0.5934), while open-source zero-shot LLMs performed markedly worse in this exploratory comparison. A 26.5% CRS gap separated the best and worst fine-tuned models. Rankings were identical under both DRG schemes (Spearman *ρ* = 1.00), indicating stability under a tiered Tw-DRG approximation (93.9% coverage), not the official grouper. At a 20% review rate, revenue-targeted prioritization achieved 43.2% CRS reduction versus 20.0% for random sampling, reaching 91% of the oracle bound. Revenue-aware evaluation captures financially meaningful differences missed by standard metrics, and revenue-guided human-AI collaboration emerges as a candidate deployment framework requiring prospective validation.

**Supplementary Information:**

The online version contains supplementary material available at 10.1038/s41598-026-57682-0.

## Introduction

The International Classification of Diseases, Tenth Revision, Clinical Modification (ICD-10-CM) serves as the global standard for encoding diagnoses in clinical settings. Healthcare systems worldwide depend on accurate ICD coding for epidemiological surveillance, clinical research, and reimbursement under Diagnosis-Related Group (DRG) payment systems^1,2^. DRG-based prospective payment has been adopted in over 30 countries^3^. In Taiwan, the National Health Insurance (NHI) program, which covers over 99% of the population^4^, adopted the Taiwan Diagnosis-Related Group (Tw-DRG) system to transition from fee-for-service to prospective payment^5^. Under this system, each hospital admission is assigned a DRG category based primarily on its ICD-10-CM codes, and the hospital receives a fixed payment determined by the DRG weight. Consequently, errors in ICD coding directly translate to financial losses or overpayments^6^.

Manual ICD coding remains labor-intensive and error-prone. Professional coders must review lengthy clinical documents, often exceeding 2000 words for discharge summaries, and assign multiple codes from a vocabulary of over 70,000 ICD-10-CM codes. Studies have reported inter-coder agreement rates as low as 60–80% for complex cases^7^, with coding error rates ranging from 3 to 30% depending on case complexity^8^. Because ICD coding is multi-label, even small per-code error rates compound across each admission’s full code set.

Deep learning methods have gained traction in automated ICD coding. Early approaches used convolutional neural networks (CNNs)^9^ with per-label attention mechanisms^10^, and subsequent work introduced multi-resolution feature extraction^11^ and label attention networks^12^. Pretrained language models such as PLM-ICD^13^, which partitions long documents into RoBERTa-processed^14^ chunks built on the transformer architecture^15^ and bidirectional-encoder pretraining^16^, achieved state-of-the-art results on MIMIC-IV. The Edin reproducibility study^17^ benchmarked six models under a unified framework, providing reliable baselines for comparison.

Large language models (LLMs) are widely used in clinical NLP^18,19^, yet perform poorly on ICD coding: even GPT-4 achieves far lower accuracy than fine-tuned models^20^, a finding attributed to the extreme label space (> 70,000 codes) and exact-code prediction requirement.

Three gaps in the current literature motivated this work. Existing evaluations use standard classification metrics (F1, AUC) that treat all coding errors as equally consequential, ignoring that coding errors occur in financial contexts of widely varying magnitude^21^. No study has compared how automated coding performance translates across national DRG systems^3^, an important question for countries like Taiwan that rely on US-derived research for technology adoption. Furthermore, existing human-AI collaboration approaches route uncertain cases to human review without considering that some errors occur in financial contexts of far greater consequence than others^22–24^.

To address these gaps, this study benchmarked 11 models under heterogeneous evaluation conditions: six Edin benchmark models on the full 7942-code ICD-10-CM label space, two self-trained baselines on the top-50 codes, and three zero-shot open-source LLMs on 200 test admissions. Because the settings differ, direct head-to-head comparability across all 11 models is limited, and cross-category comparisons are reported as exploratory rather than like-for-like benchmarking. The Revenue Sensitivity Index (RSI) quantifies the financial context of each code within a DRG system, and the Coding Reimbursement Score (CRS) aggregates code-level RSI into an admission-level reimbursement risk measure. Model performance was then compared under US MS-DRG and Taiwan Tw-DRG systems to test whether coding research conducted on US clinical data generalizes to other national healthcare contexts. A simulation-based human-AI collaboration framework was also developed in which admissions are prioritized for human review based on estimated revenue risk, exploring a candidate review-prioritization strategy to be validated prospectively before real-world deployment.

## Results

### Model performance

Table 1 presents the comprehensive model comparison. Among the six Edin benchmark models on the full 7942-code label space, PLM-ICD achieved the best F1-micro of 0.5934 (95% CI 0.591–0.595), followed by LAAT (0.5829), MultiResCNN (0.5732), CAML (0.5554), Bi-GRU (0.5035), and CNN (0.4811). This ranking was consistent across all metrics. AUC-micro scores were uniformly high (0.976–0.993), confirming strong ranking ability even where F1 scores diverged substantially. The precision-recall balance differed across architectures: LAAT achieved the highest precision (0.6172) but lower recall (0.5522), whereas PLM-ICD balanced precision (0.6149) and recall (0.5733) more evenly. CNN showed the largest precision-recall gap (0.5318 vs 0.4392), indicating that simpler architectures tend toward conservative predictions that miss more true diagnoses.

**Table 1:** Performance comparison of six Edin benchmark models on the medical information mart for intensive care IV (MIMIC-IV) international classification of diseases, tenth revision, clinical modification (ICD-10-CM) test set (19,827 admissions, 7942 codes). Best values are in bold.

Model	F1-micro	95% CI	F1-macro	AUC-micro	Precision	Recall
**PLM-ICD**	**0.5934**	0.591–0.595	**0.2329**	**0.9926**	0.6149	**0.5733**
LAAT	0.5829	0.581–0.585	0.2085	0.9909	**0.6172**	0.5522
MultiResCNN	0.5732	0.571–0.575	0.2143	0.9896	0.6053	0.5443
CAML	0.5554	0.554–0.557	0.1667	0.9859	0.5809	0.5320
Bi-GRU	0.5035	0.502–0.506	0.1227	0.9834	0.5434	0.4690
CNN	0.4811	0.479–0.483	0.0944	0.9762	0.5318	0.4392

The performance gap between PLM-ICD and the simpler architectures reflects the advantage of pretrained language representations combined with document chunking. PLM-ICD processes the full discharge summary through overlapping segments, whereas CNN and Bi-GRU models must truncate longer inputs.

The self-trained TF-IDF baseline achieved F1-micro of 0.5528 on the restricted top-50 label space, while BioClinicalBERT reached only 0.4645, underperforming the simpler baseline because its 512-token limit discards most of each discharge summary (99.7% exceeded this length). Architectural sophistication alone does not compensate for information loss: MIMIC-IV discharge summaries frequently exceed 2000 tokens. The three zero-shot LLMs fared far worse: MedGemma (F1-micro = 0.0185), BioMistral (0.0058), and TachyHealth (0.0013), consistent with prior findings that task-specific fine-tuning remains necessary for ICD coding^20^. The extreme label space renders zero-shot prompting impractical for exact-code prediction. TF-IDF achieved high recall (0.7813) but low precision (0.4278), while BioClinicalBERT showed the inverse pattern (precision 0.6954, recall 0.3487), confirming that its 512-token truncation primarily manifests as missed diagnoses.

**Fig. 1:**
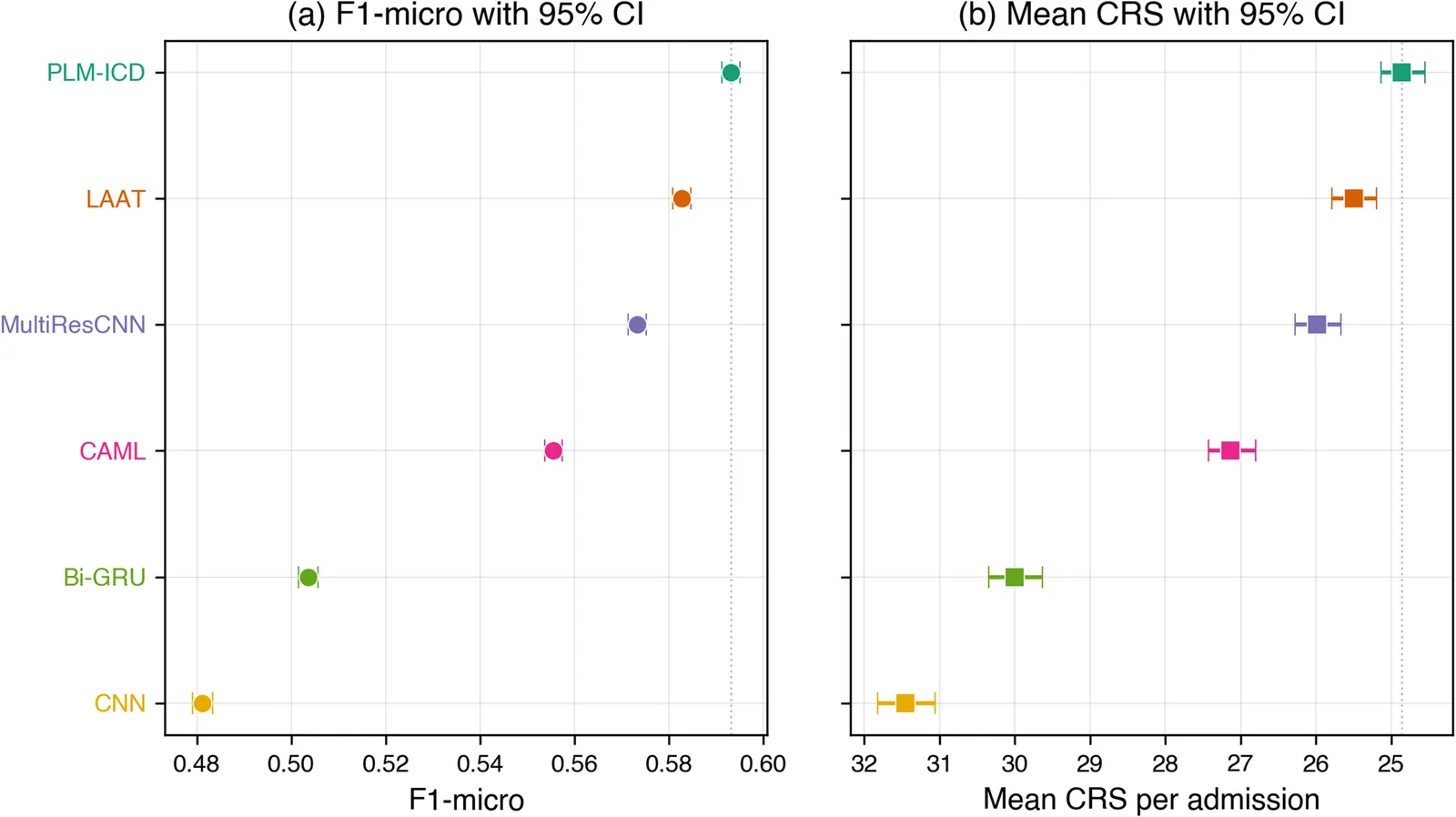
Forest plot of micro-averaged F1 (left) and mean Coding Reimbursement Score (CRS) per admission (right) with 95% bootstrap confidence intervals for the six Edin benchmark models. PLM-ICD achieves the highest F1 and lowest CRS.

All 15 pairwise comparisons among the six Edin models were statistically significant after Holm-Bonferroni correction (*P* < 0.05) for both McNemar’s test and Wilcoxon signed-rank test. The 95% bootstrap confidence intervals showed no overlap between any model pair (Fig. 1).

All six models were well calibrated^25^ (Expected Calibration Error < 0.01, range 0.0066–0.0095), with high-bin accuracy exceeding 0.98. The dissociation between calibration quality and prediction accuracy means even lower-F1 models can provide reliable probability estimates for risk stratification.

### Error characterization

The five-category error taxonomy (Table 2) revealed that over-prediction (E5) was the dominant error type across all models, accounting for 33–36% of all errors. Chapter confusion (E3) was the second most common at 26.5–28.2%, followed by complete misses (E1) at 18–23%. Better-performing models had fewer complete misses: PLM-ICD achieved the lowest E1 rate (17.7%) versus CNN’s highest (22.7%). Near-miss rates (E2) were relatively stable across models (9–11%), suggesting that sibling-code discrimination difficulty is more a property of ICD-10-CM structure than model architecture.

**Table 2:** Error taxonomy distribution (%). E1 = complete miss, E2 = near miss, E3 = chapter confusion, E4 = irrelevant prediction, E5 = over-prediction. FN: false negative; FP: false positive. Counts in thousands.

Model	E1%	E2%	E3%	E4%	E5%	FN (K)	FP (K)
PLM-ICD	**17.7**	11.4	28.2	9.6	33.1	138.4	103.1
LAAT	17.8	10.9	27.6	9.4	34.4	140.2	109.1
MultiResCNN	17.8	10.6	27.4	9.8	34.4	142.6	113.0
CAML	17.9	10.1	26.5	9.9	35.6	146.5	122.3
Bi-GRU	21.0	9.8	27.7	**7.5**	34.0	168.5	119.3
CNN	22.7	9.1	27.1	7.5	33.7	175.8	123.1

Error distributions differed significantly across models (*χ*^2^ = 6783, *P*≈0), though Cramér’s *V* = 0.033 indicated small practical effect size, consistent with models sharing fundamentally similar error patterns despite performance differences (Supplementary Fig. S1). Joining each false-positive error to the MS-DRG RSI of the mispredicted code showed that PLM-ICD’s 79,985 E5 over-prediction instances were distributed almost uniformly across RSI quartiles (Q1 24.3%, Q2 24.4%, Q3 25.4%, Q4 25.9%; mean RSI 2.44 vs a baseline gold-code mean of 2.42), indicating that E5 over-prediction is not preferentially concentrated on high-RSI codes. The slight upper-quartile excess nonetheless places approximately 20,700 over-predictions per model into the highest RSI quartile, a non-trivial audit-exposure volume even without disproportionate targeting. In contrast, E4 irrelevant false positives were skewed toward low RSI (Q1 32.1%, Q4 24.7%; mean RSI 2.37) (Supplementary Fig. S8).

### Revenue impact

The CRS framework exposed wide variation in the financial context of coding errors. Across 7753 analyzable codes, the RSI distribution had a mean of 2.64 (median 2.14, SD 1.90, range 0.52–28.17) under MS-DRG. Model CRS rankings mirrored F1-micro rankings (Table 3): PLM-ICD had the lowest mean CRS (24.86 per admission) and CNN the highest (31.45), a 26.5% gap in aggregate reimbursement risk.

**Table 3:** Revenue impact and dual diagnosis-related group (DRG) system comparison.

Model	F1-micro	MS-DRG CRS (95% CI)	Tw-DRG CRS	NTD Risk (B)
**PLM-ICD**	**0.5934**	**24.86** (24.55–25.14)	**7.91**	8.4
LAAT	0.5829	25.50 (25.19–25.79)	8.11	8.6
MultiResCNN	0.5732	25.99 (25.67–26.28)	8.29	8.8
CAML	0.5554	27.14 (26.81–27.43)	8.66	9.2
Bi-GRU	0.5035	30.00 (29.63–30.34)	9.51	10.1
CNN	0.4811	31.45 (31.05–31.82)	9.93	10.6

CRS: Coding Reimbursement Score; MS-DRG: Medicare Severity DRG; Tw-DRG: Taiwan DRG; NTD: New Taiwan Dollar; SPR: standard point rate; RW: relative weight. NTD risk in billions at SPR = 53,632 NTD/RW.

The relationship between model F1-micro and mean CRS showed a strong inverse correlation, confirming that classification accuracy directly translates to financial performance (Supplementary Fig. S2). The right-skewed RSI distribution (median 2.14, IQR 1.61–3.06, max 28.17) means errors on high-RSI codes occur in disproportionate financial contexts that standard F1 metrics fail to capture. CRS rankings remained perfectly stable (Spearman *ρ* = 1.0) across all methodological variations tested: FP weight (*α* 0.0–1.0), DRG weighting scheme (uniform, mean, admission-specific), and RSI formulation (raw vs ratio). PLM-ICD maintained rank 1 across all probability thresholds (0.2–0.6) (Supplementary Fig. S3).

In aggregate across 16,677 MS-DRG-eligible test admissions, total CRS ranged from 414,593 (PLM-ICD) to 524,543 (CNN), a difference of 109,950 CRS points. Under the Tw-DRG system with the NHI standard point rate of NTD 53,632 per relative weight, the estimated coding risk ranged from NTD 8.41 billion (PLM-ICD) to NTD 10.56 billion (CNN) across all 19,827 test admissions. The NTD 2.15 billion gap from a single institution illustrates how per-admission CRS differences accumulate into system-level financial consequences.

### Dual DRG system analysis

Model CRS rankings under MS-DRG and the Tw-DRG approximation were identical (Spearman *ρ* = 1.00). All six models maintained the same ranking positions under both weight schemes: PLM-ICD < LAAT < MultiResCNN < CAML < Bi-GRU < CNN (Table 3). Under the tiered Tw-DRG approximation applied here (93.9% admission coverage, not the official Tw-DRG grouper), model relative rankings derived from MIMIC-IV were preserved when admissions were reweighted with Taiwan payment weights.

**Fig. 2:**
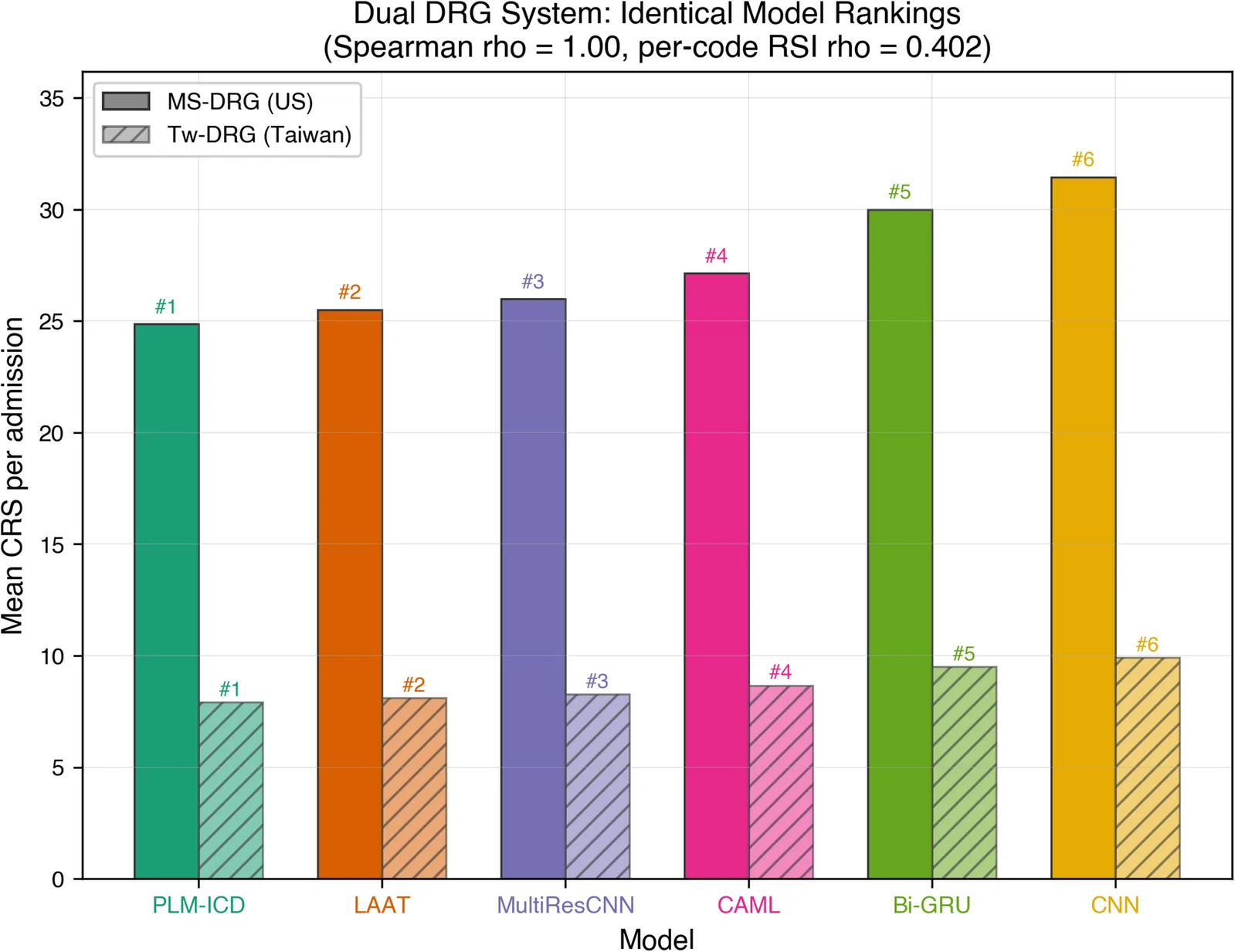
Dual diagnosis-related group (DRG) system comparison: mean coding reimbursement score (CRS) per admission under medicare severity DRG (MS-DRG; US) and Taiwan DRG (Tw-DRG; Taiwan). Despite threefold difference in absolute CRS scale, model rankings are identical (Spearman rho = 1.00).

Per-code RSI correlation between the two systems was, however, only moderate (*ρ* = 0.402, *n* = 7753). The two systems assign different financial weight to specific conditions; for example, post-procedural complication codes had higher RSI under MS-DRG, while certain chronic disease codes carried more weight in Tw-DRG. This divergence reflects differences in how the two systems define DRG groupings and assign payment weights: MS-DRG uses a severity-stratified system with complication/comorbidity tiers, whereas Tw-DRG employs a flatter structure adapted to Taiwan’s single-payer context. Aggregation from code to admission to model level smooths out per-code differences, explaining why model rankings agree perfectly despite moderate code-level correlation (Fig. 2). This finding indicates that, under the tested Tw-DRG approximation, model relative rankings were stable across the two DRG weight schemes. Direct transferability to real-world Taiwan hospital deployment would require validation with the official Tw-DRG grouper and prospective evaluation on Taiwanese clinical data, which is beyond the scope of this MIMIC-IV-based study.

### DRG grouping sensitivity analysis

To assess whether RSI reflects direct causal influence on DRG assignment or merely co-occurrence with high-weight admissions, a leave-one-out analysis was conducted on 20 target diagnosis codes: 10 selected by highest RSI and 10 by highest financial exposure (RSI multiplied by frequency). For each code appearing as a secondary diagnosis, the code was removed and the admission re-grouped through the CMS MS-DRG v42.1 and Tw-DRG groupers while holding all other variables constant. Only 6 of 20 codes produced any DRG reclassification under MS-DRG, with change rates from 3.6% (N17.9, acute kidney failure; n = 2988) to 72.7% (I51.1, septal defect; n = 11). Under Tw-DRG, a partially overlapping set of 5 codes triggered changes (4.4% to 29.4%). The rank correlation between RSI and actual DRG change rate was weak and non-significant (MS-DRG: Spearman *ρ* = 0.384, *P* = 0.095; Tw-DRG: *ρ* = 0.146, *P* = 0.538). High-RSI codes such as J95.851 (ventilator-associated pneumonia, RSI = 8.41, n = 114) produced zero DRG changes upon removal, whereas moderate-RSI codes like D62 (acute posthemorrhagic anemia, RSI = 3.77, n = 1565) triggered 8.6% MS-DRG reclassifications. These results confirm that RSI captures the financial context of admissions in which a code appears rather than the code’s capacity to shift DRG assignment (Supplementary Fig. S5).

### Human-AI collaboration

**Fig. 3:**
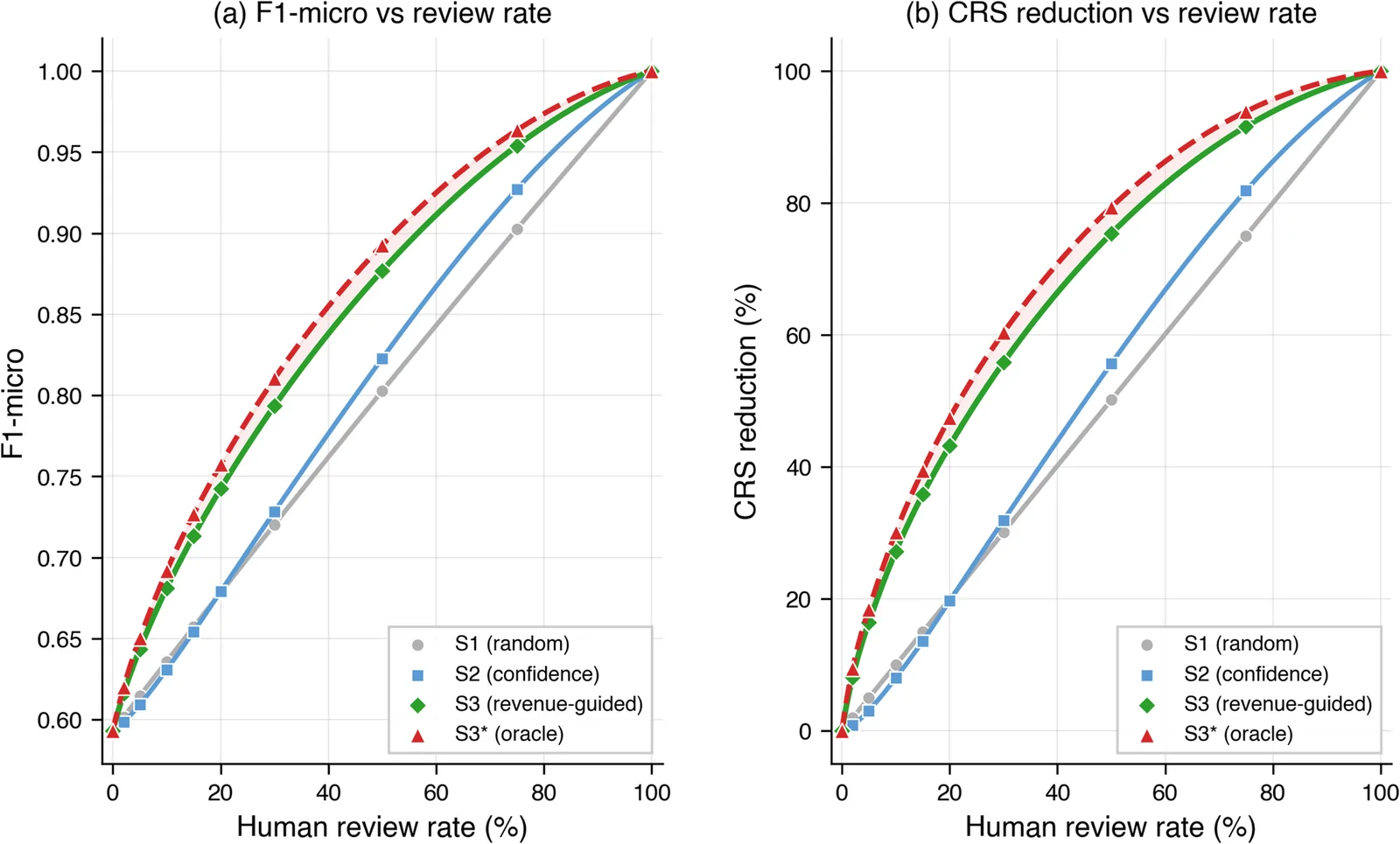
Human-AI collaboration strategies for PLM-ICD: micro-averaged F1 (left) and Coding Reimbursement Score (CRS) reduction (right) versus human review rate. The revenue-guided strategy (S3) outperforms random (S1) and confidence-based (S2) approaches at all review rates while approaching the oracle upper bound (S3*). S3-TW (Tw-DRG variant) is omitted because its values are nearly identical to S3 (maximum difference 0.35%).

Revenue-targeted review (S3) outperformed all alternatives at every review rate tested (Table 4, Fig. 3). At the practically relevant 20% review rate: S3 achieved F1-micro of 0.7423 with 43.2% CRS reduction; S3-TW (Tw-DRG targeting) achieved similar F1 of 0.7435 with 43.1% CRS reduction; while S2 (confidence-based) achieved only 19.7% and S1 (random) achieved 20.0% CRS reduction. The practical S3 strategy attained 91% of the theoretical oracle upper bound (43.2% vs 47.4%).

**Table 4:** Human-AI collaboration simulation results for PLM-ICD. CRS: Coding Reimbursement Score. CRS reduction (%) is relative to the no-review baseline.

Strategy	F1 (10%)	F1 (20%)	F1 (50%)	CRS Red. 10%	CRS Red. 20%	CRS Red. 50%
S1 (random)	0.636	0.678	0.803	10.0	20.0	50.1
S2 (confidence)	0.631	0.679	0.823	8.0	19.7	55.6
S3 (revenue, MS-DRG)	0.681	**0.742**	0.877	27.2	**43.2**	75.4
S3-TW (revenue, Tw-DRG)	0.682	0.744	0.878	27.1	43.1	75.0
S3* (oracle)	0.692	0.757	0.893	30.1	47.4	79.4

The near-equivalence of MS-DRG and Tw-DRG prioritization (S3 vs S3-TW) suggests either system can guide review prioritization with minimal loss of effectiveness. The failure of confidence-based review (S2) to outperform random sampling indicates that model uncertainty is a poor proxy for financial risk.

The gap between S3 and the oracle (S3*) narrowed as the review rate increased: at 10% review, S3 captured 90% of the oracle benefit (27.2% vs 30.1%); at 20%, 91% (43.2% vs 47.4%); and at 50%, 95% (75.4% vs 79.4%). This convergence indicates that the estimated CRS accurately identifies the highest-risk admissions, with diminishing room for improvement as more admissions are reviewed. Cost-effectiveness analysis (Supplementary Fig. S4) identified a 10–20% review rate as the simulation-based optimum pending prospective validation, with marginal returns diminishing sharply above 20%.

Because the primary simulation assumed perfect human correction, a sensitivity analysis was conducted in which each AI error in a reviewed admission was corrected with probability *1—e* for realistic reviewer error rates *e* ∈ {10%, 20%, 30%}^8^. CRS reduction degraded approximately linearly with *e*: at the 20% review rate, S3 achieved 43.2%, 38.9%, 34.6%, and 30.2% CRS reduction under MS-DRG for *e* = 0%, 10%, 20%, 30% (F1-micro 0.742, 0.728, 0.713, 0.698), and S3-TW showed a parallel degradation under Tw-DRG (38.3%, 34.5%, 30.7%, 26.8%). Even at the highest reviewer error rate (30%), revenue-targeted review retained 30.2% CRS reduction under MS-DRG, still 1.5-fold greater than random sampling at perfect correction (20.0%). The framework thus tolerates realistic reviewer imperfection while preserving most of its simulated advantage over untargeted review (Supplementary Fig. S6). This sensitivity model captures failure to correct AI errors only; human-introduced new miscoding of previously-correct codes is an additional error source not modelled here and is expected to further attenuate benefit in prospective deployment.

## Discussion

PLM-ICD achieved the highest F1-micro (0.5934) on the full 7942-code space, while the three open-source zero-shot LLMs showed poor performance on a 200-admission exploratory sample; this latter observation is preliminary and may not generalize to frontier or fine-tuned LLMs. The RSI/CRS framework showed that coding errors occur in financial contexts of widely different magnitude: the 11.2 percentage-point F1 gap between PLM-ICD and CNN translated to a 26.5% difference in reimbursement risk. Model quality rankings were identical under MS-DRG and the Tw-DRG approximation (*ρ* = 1.00), indicating that ranking stability is preserved under the tested Taiwan-weighted re-evaluation of MIMIC-IV admissions. Finally, revenue-targeted review at 20% achieved more than double the CRS reduction of random sampling and reached 91% of the oracle upper bound.

F1-micro results for the six Edin models align with the original benchmark^17^; the main contribution here is the revenue-sensitive evaluation, dual DRG analysis, and human-AI simulation. The LLM results align with previous findings^20^, but were obtained on a much smaller sample (200 admissions) and a different evaluation setup than the full-space Edin models. The present study therefore offers an exploratory observation that open-source zero-shot LLMs performed very poorly in this setting, rather than a definitive quantitative comparison on equal footing; results could differ for alternative, more advanced, or fine-tuned LLMs. Prior automated coding studies focused on classification accuracy^10,11,21^; none quantified financial risk via DRG-weighted metrics or compared coding impact across national DRG systems.

Uniformly high AUC-micro scores (0.976–0.993) coexist with a wide F1-micro range (0.481–0.593): with 7942 codes per admission, the vast majority of code-admission pairs are true negatives that all models correctly identify. F1-micro, threshold-dependent, better reflects operational coding performance and motivates revenue-sensitive metrics over threshold-insensitive measures.

The magnitude of these financial differences warrants emphasis. From a hospital administrator’s perspective, the 26.5% gap in reimbursement risk between the best and worst models is a far more actionable metric than the corresponding 11.2 percentage-point F1 gap. Unlike F1-score, which requires statistical literacy to interpret, CRS provides a revenue-weighted summary of coding error magnitude that can be expressed in DRG-based monetary units, while remaining an association-based indicator rather than a direct forecast of reimbursement impact. The framework is portable: RSI can be computed for any DRG-based payment system given a grouper and weight table, supporting health technology assessment (HTA) and procurement decisions that require cost-effectiveness evidence.

The leave-one-out DRG grouping analysis provides additional context for interpreting RSI. The weak correlation between RSI rank and actual DRG change rate (Spearman *ρ* = 0.384 for MS-DRG, *ρ* = 0.146 for Tw-DRG; both non-significant) confirms that RSI is not a measure of direct causal influence on DRG assignment. Instead, it quantifies the financial milieu in which codes co-occur. This raises a natural question: if removing a high-RSI code rarely changes DRG assignment on its own, through what mechanisms do coding errors translate into financial consequences? Four non-exclusive mechanisms are relevant. First, portfolio accumulation: because CRS uses RSI as a weighting factor across all missed or extra codes per admission, it functions as a population-level risk metric that accumulates many small contributions rather than depending on any single code’s DRG-shifting capacity. Second, co-occurrence effects: multiple concurrent coding errors on the same admission may jointly cross a DRG boundary that no single-code change would produce, an interaction that single-code leave-one-out cannot detect. Third, complication/comorbidity (CC) and major CC (MCC) threshold crossings: specific codes that serve as the CC or MCC required to upgrade a severity tier carry DRG-shifting impact at the margin, which is why 6 of 20 tested codes produced some MS-DRG reclassification and a subset produced very high change rates (up to 72.7% for I51.1). Fourth, audit exposure: over-prediction errors generate upcoding risk and downstream repayment or regulatory liabilities even when they do not directly change DRG assignment. CRS aggregates the first two mechanisms by design; the third is visible in the leave-one-out tails; the fourth operates outside the DRG-weight calculation but contributes to the regulatory significance of the dominant over-prediction error type (E5). The sensitivity analysis thus validates RSI’s intended role as a population-level financial indicator while clarifying that it should not be interpreted as measuring individual codes’ DRG-shifting power.

Confidence-based review (S2) was no better than random sampling for CRS reduction. Model uncertainty does not track financial risk: well-calibrated confidence identifies codes where the model is uncertain, not codes where errors are financially consequential. The good calibration across all models (ECE < 0.01) confirms that confidence estimates are statistically reliable; the problem is that they measure the wrong quantity for financial risk management.

At a 20% review rate, all six models achieved S3 CRS reductions within a narrow range (42.0–43.5%), while S2 achieved only 19.1–21.8% (barely above random). The tight clustering suggests the benefit derives from the prioritization strategy rather than model quality. Even CNN (F1 = 0.48) achieved 42.0% CRS reduction with revenue-targeted review versus 20.0% for random; if confirmed prospectively, hospitals constrained from deploying the best model may still capture substantial financial benefit through intelligent review allocation.

The identical cross-system rank correlation supports the more limited view that, under the Tw-DRG approximation tested here, model relative rankings derived from US data remain stable when admissions are reweighted with Taiwan payment values^26^; whether this stability extends to real-world Taiwan hospital deployment would require validation with the official Tw-DRG grouper on local clinical data. Under Tw-DRG with the standard point rate of NTD 53,632, PLM-ICD generates lower coding risk than the alternatives. For a typical Taiwan hospital processing 50,000 DRG-eligible admissions annually, even modest improvements in coding accuracy, amplified by revenue-aware prioritization, could translate into meaningful financial savings. The mean Tw-DRG RSI (0.87) was approximately one-third of the MS-DRG mean (2.64), reflecting the generally lower payment weights in Taiwan’s single-payer system, yet model performance rankings were perfectly preserved despite this three-fold difference in absolute scale. The revenue-targeted review strategy using Tw-DRG weights (S3-TW) achieved 43.1% CRS reduction at a 20% review rate in the simulation, nearly identical to the MS-DRG-based S3 (43.2%), suggesting that the revenue-targeted framework could be piloted in Taiwan using locally computed Tw-DRG RSI values, subject to prospective validation with the official Tw-DRG grouper. Candidate implementation directions for such prospective studies, rather than validated deployment guidance, include: (1) PLM-ICD or comparable PLM-based model as a primary automated coder; (2) a revenue-targeted review queue using Tw-DRG CRS estimates to prioritize admissions; (3) an adaptive review rate in the 10–20% range calibrated to available human coder capacity; and (4) continuous calibration monitoring. The RSI/CRS framework is general and can be applied to other national DRG systems, enabling cross-national comparisons. Integrating revenue-sensitive loss functions into model training could steer gradient updates toward high-RSI codes. Future prospective studies should evaluate the framework with real human coders to measure correction rates and inter-coder variability under time constraints; longitudinal deployment studies could clarify whether periodic recalibration is needed.

Over-prediction (E5, 33–36%) dominates across all models, identifying threshold calibration as the most promising near-term improvement. Near-miss rates (E2, 9–11%) are stable, suggesting sibling-code discrimination is an inherent ICD-10-CM challenge. Better models primarily reduce complete misses (E1). Across models, unique codes in false-positive predictions (3612–4632) were far fewer than in false negatives (7765–7914), so models over-predict from a concentrated set while missing a broader range of true diagnoses. Improving recall for rare high-RSI codes offers the greatest opportunity for reducing CRS.

Beyond financial consequences, coding errors affect quality measurement: under-coding (E1) distorts hospital case-mix indices used for resource allocation and quality benchmarking, and over-coding (E5, 33–36%) raises audit-exposure concerns under Taiwan’s NHI claims-review regime. The E5 × RSI cross-tabulation (Supplementary Fig. S8) shows this exposure arises from volume rather than preferential targeting of high-RSI codes: E5 mean RSI tracks baseline (2.44 vs 2.42), so audit risk scales with total excess-code count. The revenue-targeted review framework directs human attention to admissions where coding errors occur in financial contexts of greatest magnitude and regulatory salience.

This work has several limitations. MIMIC-IV originates from a single US tertiary academic medical center, Beth Israel Deaconess Medical Center in the Boston metropolitan area, whose patient population may skew toward higher acuity and more complex cases than those of most US community hospitals. Because earlier MIMIC releases were restricted to intensive-care admissions, ICU-origin patients may remain over-represented in MIMIC-IV relative to a general-hospital case mix. A comparison of the MIMIC-IV test split (17,225 admissions) with CMS national Medicare claims (FY 2023 V42 grouper, 6,871,447 claims) showed that the distributions, while strongly correlated (Spearman *ρ* = 0.846), differ in top-20 concentration (MIMIC-IV 23.9% vs CMS 36.2%) and over-represent specialty and tertiary-care DRGs (chemotherapy DRG 847, heart failure with complications DRG 292, coronary bypass DRG 236) in MIMIC-IV (Supplementary Fig. S7). Institution-specific staffing patterns, documentation conventions, coding practices, billing resources, and technology infrastructure further limit generalizability to broader US hospital settings, and transfer to non-US healthcare systems (including Taiwan) should not be assumed from this dataset alone. Taiwan additionally uses a localized version of ICD-10-CM with additional codes not present in the evaluation, and its Tw-DRG groupings are not captured by the tiered approximation (93.9% coverage) used here in place of the official Tw-DRG grouper. The primary human-AI collaboration simulation assumes perfect human correction; the sensitivity analysis reported above (Supplementary Fig. S6) quantifies how the simulated benefit decreases approximately linearly with reviewer error rate, but does not replace prospective validation with real coders. The FP weight *α* = 0.5 is a modeling choice, though sensitivity analysis confirmed rank stability across all *α* values tested. Finally, all evaluations were retrospective; prospective validation in a live coding environment is needed to assess real-world deployment effectiveness.

The financial contexts surrounding coding errors vary by more than 50-fold depending on the clinical context and DRG system, a dimension that standard classification metrics miss entirely. In this simulation, model quality rankings were consistent across the two DRG weight schemes tested, and revenue-targeted review of 20% of admissions reduced reimbursement-weighted error magnitude (CRS) by 43%. Together, the revenue-sensitive evaluation framework and the human-AI collaboration simulation presented here outline candidate directions for future prospective deployment studies of AI-assisted ICD-10-CM coding in DRG-based healthcare systems.

## Methods

### Ethical considerations

This study used the publicly available MIMIC-IV dataset, which contains de-identified health records. Access was obtained through PhysioNet after completing the required data use agreement and human subjects training. No additional institutional review board approval was required.

### Study design

**Fig. 4:**
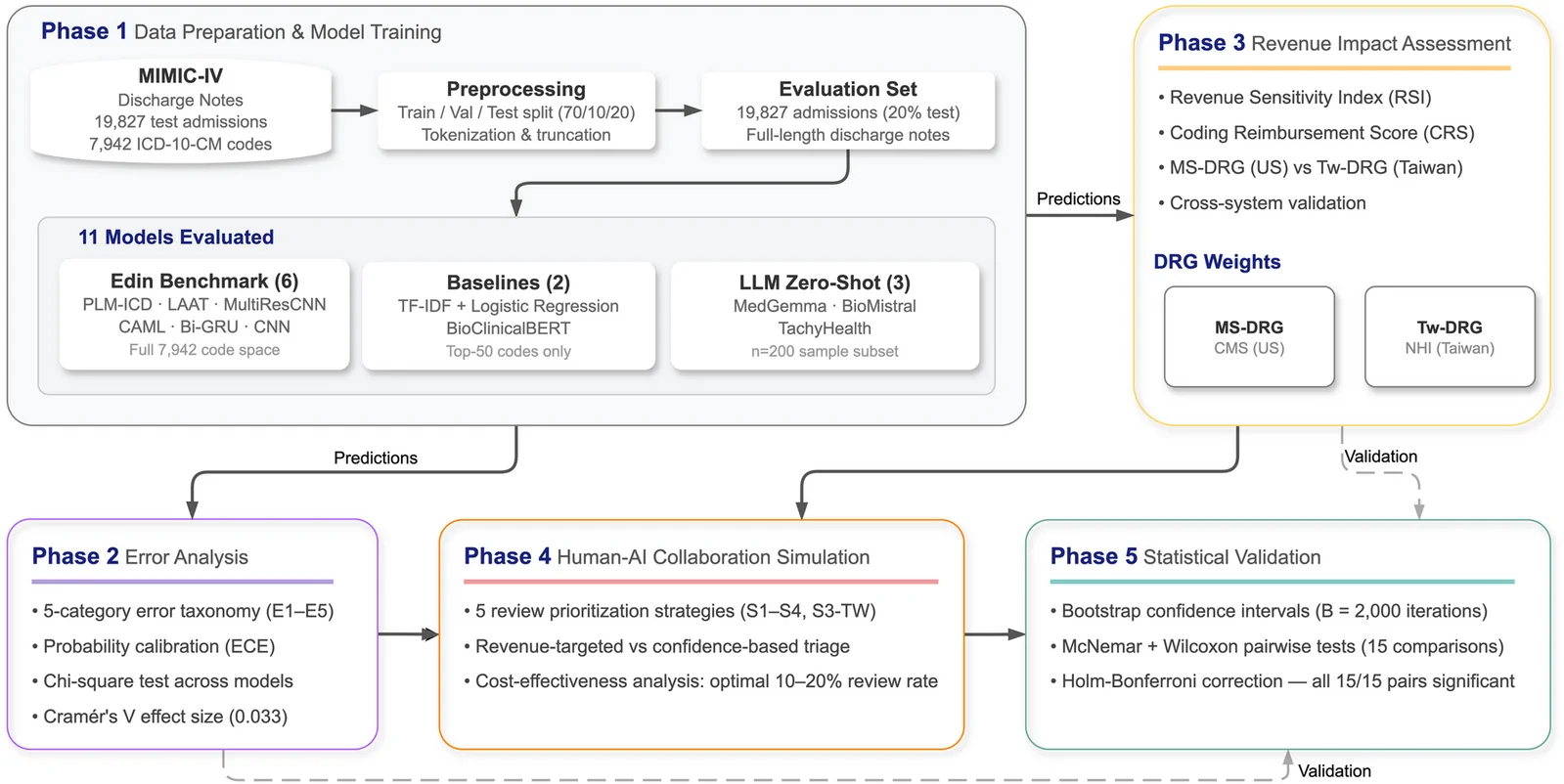
Research pipeline: five-phase analysis from data preparation and model training through error taxonomy, revenue impact assessment under dual Diagnosis-Related Group (DRG) systems, human-AI collaboration simulation, and statistical validation.

This is a retrospective multi-model evaluation study using discharge summaries from the MIMIC-IV database to benchmark automated ICD-10-CM coding performance and assess financial impact under two DRG payment systems. Figure 4 illustrates the overall research pipeline.

### Data sources

Two publicly available datasets from the MIMIC (Medical Information Mart for Intensive Care) project^27,28^ were used: (1) MIMIC-IV v2.2, containing de-identified health records from Beth Israel Deaconess Medical Center spanning 2008–2019, from which hospital admissions, ICD-10-CM diagnosis codes, and DRG assignments were extracted; and (2) MIMIC-IV-Note v2.2, providing discharge summaries as input text for coding models.

Following the Edin benchmark protocol^17^, analysis was restricted to admissions with ICD-10-CM codes (version 10) and valid discharge summaries, yielding 99,134 admissions coded with 7942 unique ICD-10-CM codes. A chronological train/validation/test split was adopted: 69,393 training (70%), 9914 validation (10%), and 19,827 test admissions (20%). The mean number of codes per admission was 15.8 (median 14, range 1–154).

For revenue analysis, DRG data were integrated from two sources. MS-DRG assignments and relative weights were extracted from MIMIC-IV^29^ (16,677 test admissions with valid MS-DRG, 84.1%). Tw-DRG weights were constructed by mapping ICD-10-CM codes to Taiwan’s Tw-DRG classification using the official NHI weight table^5^ through a tiered approach. Tier 1 applied direct ICD-to-Tw-DRG mapping using the official classification table, achieving 67.7% code coverage. For codes not directly mapped in Tier 1, Tier 2 assigned weights based on the Major Diagnostic Category (MDC) chapter-level mean, raising combined coverage to 93.9%. A third tier assigned MDC-level average weights to the remaining 6.0% of admissions, and only 26 admissions (0.13%) remained unmapped. This tiered strategy balances precision (direct mapping where available) with coverage (chapter-level and MDC-level fallback for unmapped codes).

## Models

Eleven models across three categories were evaluated.

### Self-trained baselines

Two models were trained on the top 50 most frequent ICD-10-CM codes: (1) **TF-IDF + Logistic Regression**, using unigrams and bigrams with 10,000 features and L2 regularization; and (2) **BioClinicalBERT**^30,31^, fine-tuned with a multi-label classification head, limited to 512 tokens.

### Edin benchmark models

Six models from the Edin benchmark^17^ operated on the full 7942-code label space: **CNN**^32^, a convolutional network with max-pooling; **Bi-GRU**^33^, a bidirectional gated recurrent unit network; **CAML**^10^, combining CNNs with per-label attention; **MultiResCNN**^11^, using multiple filter sizes with residual connections; **LAAT**^12^, a label attention model with joint text-label embeddings; and **PLM-ICD**^13^, using RoBERTa with document chunking for long-text processing.

### LLM zero-shot

Three open-source LLMs were evaluated in zero-shot mode on 200 random test admissions: **MedGemma 4B** (Google), **BioMistral-7B**, and **TachyHealth**. Each model received the discharge summary text and was prompted to generate ICD-10-CM codes.

## Evaluation metrics

### Standard metrics

Following standard multi-label evaluation practice^34^, micro-averaged F1 (F1-micro), macro-averaged F1 (F1-macro), micro- and macro-averaged AUC-ROC, and micro-averaged precision and recall were reported. Probability thresholds were optimized on the validation set.

### Revenue sensitivity index

The RSI quantifies the financial context associated with each ICD-10-CM code within a DRG system:

1 $${\mathrm{RSI}}\left( c \right) = \overline{w}\left( c \right)/\overline{w}\left( {{\mathrm{global}}} \right)$$

where w(*c*) is the mean DRG relative weight across admissions containing code *c* and *w*(global) is the global mean DRG weight. An RSI > 1 indicates higher-than-average reimbursement association. The rationale for using the mean DRG weight rather than a causal attribution is that ICD codes influence DRG assignment through complex grouper logic that depends on the full set of codes per admission; RSI captures the empirical association between a code’s presence and the financial magnitude of the admissions in which it appears, without assuming a direct causal pathway from individual code to DRG assignment.

### Coding reimbursement score

The CRS aggregates code-level RSI into an admission-level reimbursement risk measure:

2 $${\mathrm{CRS}}\left( a \right) = w\left( a \right) \times \left[ {\mathop \sum \limits_{{c \in {\mathrm{FN}}\left( a \right)}} {\mathrm{RSI}}\left( c \right) + \alpha \times \mathop \sum \limits_{{c \in {\mathrm{FP}}\left( a \right)}} {\mathrm{RSI}}\left( c \right)} \right]$$

where w(*a*) is the DRG relative weight, FN(*a*) and FP(*a*) are false negative and false positive code sets, and *α* = 0.5 reflects the asymmetry between under-coding (direct revenue loss) and over-coding (audit risk). The asymmetric weighting is motivated by the observation that missed codes (false negatives) represent potential revenue loss, as under-coding may contribute to DRG downgrading when the missing code is a complication or comorbidity modifier, whereas extra codes (false positives) pose probabilistic audit risk that may or may not result in financial penalty. Lower CRS indicates better financial performance.

Sensitivity analyses varied the FP weight (*α* from 0.0 to 1.0), DRG weighting scheme (uniform, mean, admission-specific), and RSI formulation (raw absolute weights vs ratio), measuring rank stability via Spearman’s correlation across all six models. Probability threshold sensitivity was additionally assessed at thresholds 0.2–0.6.

### Dual DRG system analysis

RSI and CRS were computed under both MS-DRG and Tw-DRG independently. For the main revenue analysis, Tw-DRG case weights were assigned via a tiered approximation mapping from MS-DRG (93.9% admission coverage) rather than the official Tw-DRG grouper; the official Tw-DRG v3.4 grouper was used only in the leave-one-out DRG sensitivity analysis described below. Cross-system comparison measured (1) Spearman’s *ρ* between model CRS rankings and (2) per-code RSI correlation across 7753 codes present in both systems. The NHI standard point rate of NTD 53,632 was used for Tw-DRG monetary conversion; this conversion provides an order-of-magnitude financial context for interpretation rather than a causal forecast of reimbursement impact for individual admissions.

### DRG grouping sensitivity analysis

A leave-one-out analysis tested whether RSI reflects direct causal influence on DRG assignment. Twenty target diagnosis codes were selected: the 10 highest by RSI and 10 highest by financial exposure (RSI multiplied by frequency). For each target code appearing as a secondary diagnosis in a test admission, the code was removed and the admission re-grouped through both the official CMS MS-DRG v42.1 Java grouper and the Tw-DRG v3.4 grouper, holding patient demographics (sex, age), principal diagnosis, remaining secondary diagnoses, and procedure codes constant. DRG change rates were computed per code, and Spearman’s rank correlation assessed the relationship between RSI rank and actual DRG change rate.

### Error taxonomy

A five-category taxonomy was developed based on ICD-10-CM hierarchy: E1 (complete miss, no hierarchical relationship with any true code), E2 (near miss, sibling within the same 3-character category), E3 (chapter confusion, same ICD-10 chapter but different 3-character category), E4 (irrelevant prediction, false positive unrelated to any true code), and E5 (over-prediction, false positive sharing chapter or category with a true code).

### Human-AI collaboration simulation

The simulation framework evaluated human review benefit at rates *r* ∈ {0, 2, 5, 10, 15, 20, 30, 50, 75, 100}%. For reviewed admissions, model predictions were replaced with ground-truth codes (simulating perfect human correction). Five prioritization strategies were compared: S1 (random baseline, averaged over 20 trials), S2 (lowest model confidence), S3 (highest estimated MS-DRG CRS), S3-TW (highest Tw-DRG CRS), and S3* (oracle using actual CRS).

### Statistical analysis

Pairwise model comparisons used McNemar’s test^35^ for element-level F1 and Wilcoxon signed-rank test^36^ for admission-level CRS. Bootstrap resampling (*B* = 2000) provided 95% confidence intervals^37^. Holm-Bonferroni correction^38^ controlled family-wise error rate across 15 pairwise comparisons (*α* = 0.05). Error distribution differences were tested with Pearson’s chi-square test, reporting Cramér’s *V* as effect size.

## Supplementary Information

Below is the link to the electronic supplementary material.

Supplementary Material 1.

## Data Availability

The MIMIC-IV dataset is available through PhysioNet (https://physionet.org/content/mimiciv/2.2/) upon completion of a data use agreement. The Tw-DRG weight table is publicly available from Taiwan’s Bureau of National Health Insurance. Analysis code will be made available upon publication.
