# Supplementary material for: Revenue-sensitive evaluation of AI-assisted ICD-10-CM coding and human-AI collaboration under dual DRG payment systems
Source: Sci Rep. 2026 Jun 11;16:26729. doi: 10.1038/s41598-026-57682-0 (PMC13507165; doi:10.1038/s41598-026-57682-0)
Supplement: Supplementary file 1 [file 41598_2026_57682_MOESM1_ESM.pdf]

Supplementary Information: Revenue-sensitive evaluation of AI-assisted ICD-10-CM coding and human-AI collaboration under dual DRG payment systems

Ming-I Chen[1], Ying-Lin Hsu[2], Yi-Hsin Chen[2,3,4]

[1]Doctoral Program in Big Data Analytics for Industrial Applications, National Chung Hsing University, Taichung, Taiwan; [2]Department of Applied Mathematics and Institute of Statistics, National Chung Hsing University, Taichung, Taiwan; [3]Department of Nephrology, Taichung Tzu Chi Hospital, Taichung, Taiwan; [4]School of Medicine, Tzu Chi University, Hualien, Taiwan

Supplementary Figures

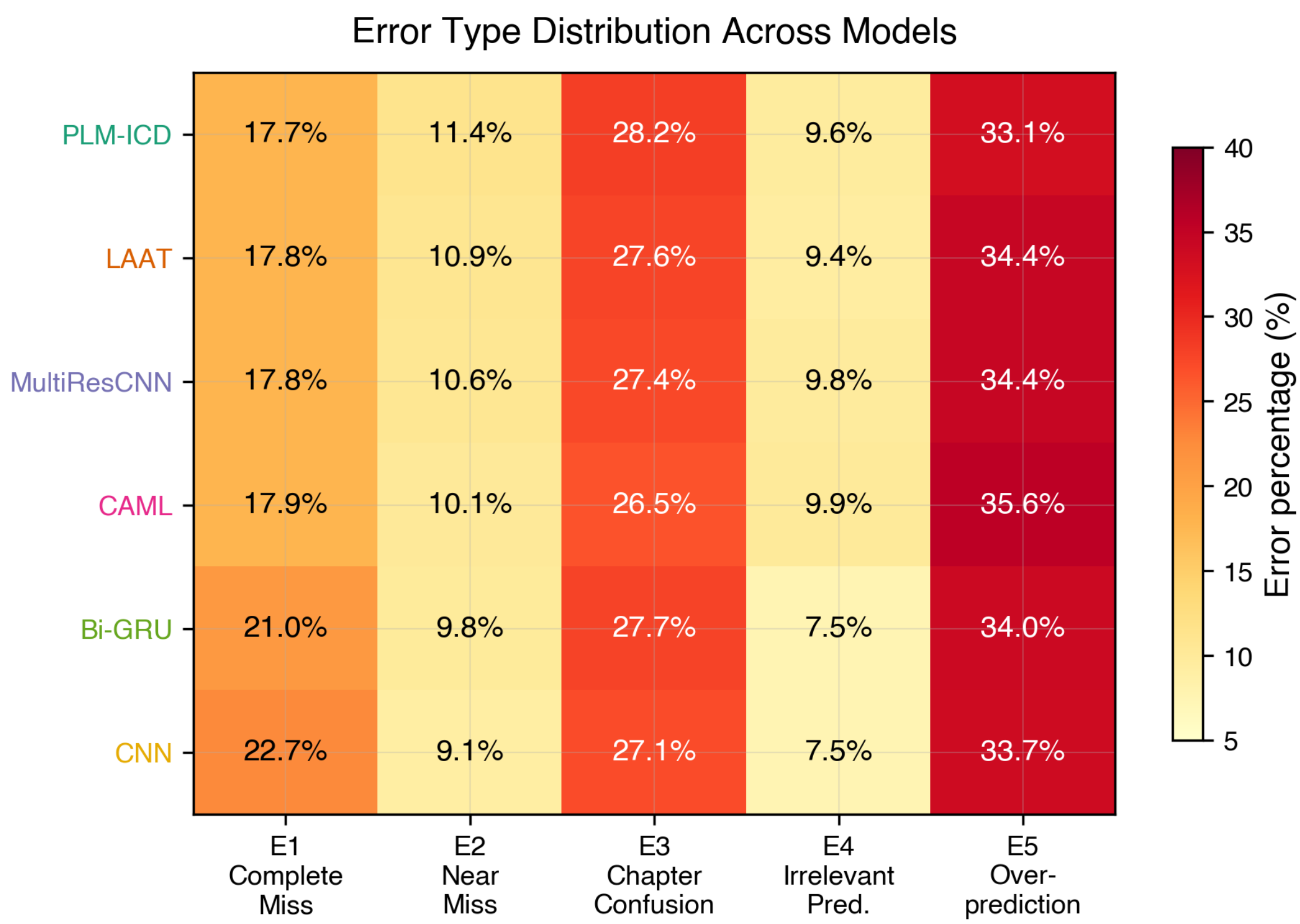

Figure 1: **Supplementary Figure S1.** Error type distribution heatmap across six models. E5 (over-prediction) dominates at 33%-36%, while E1 (complete miss) shows the largest variation by model quality (17.7%-22.7%). E1: complete miss; E2: near miss; E3: chapter confusion; E4: irrelevant prediction; E5: over-prediction.

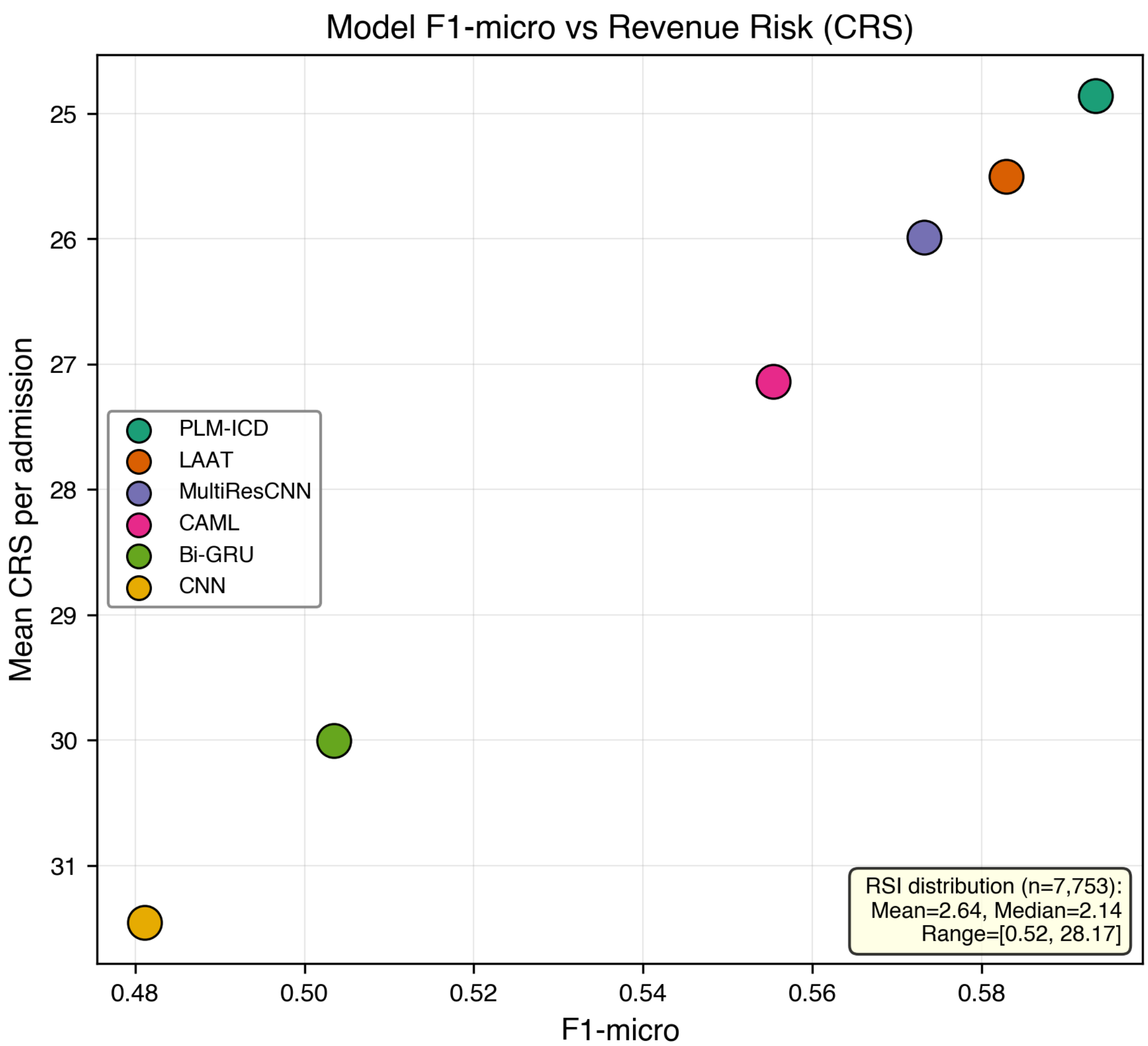

Figure 2: **Supplementary Figure S2.** Model micro-averaged F1 versus mean Coding Reimbursement Score (CRS) per admission. Higher F1 consistently corresponds to lower revenue risk. The Revenue Sensitivity Index (RSI) distribution summary is shown in the inset. CRS: Coding Reimbursement Score; RSI: Revenue Sensitivity Index.

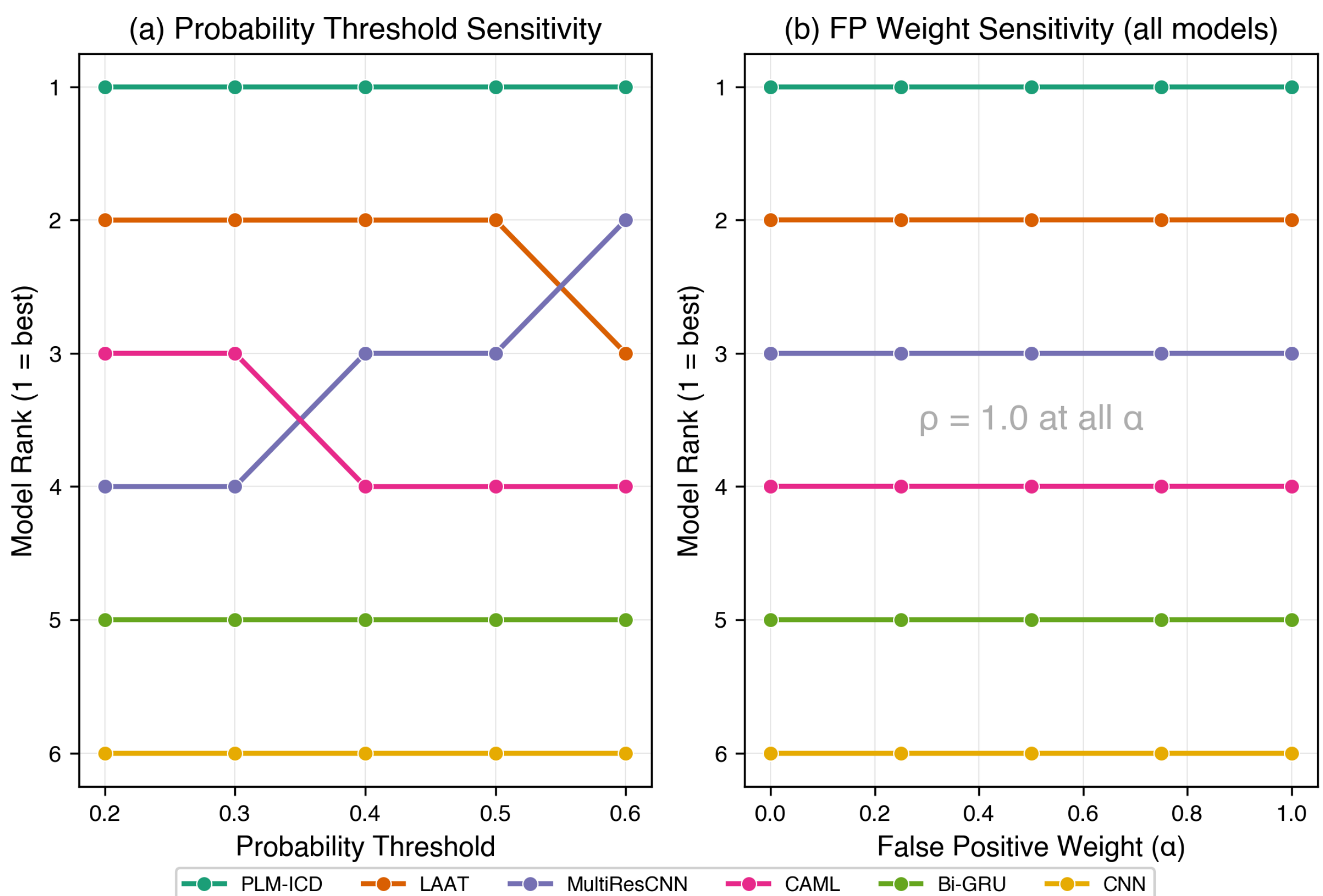

Figure 3: **Supplementary Figure S3.** CRS sensitivity analysis. Panel (a) shows model rank stability across probability thresholds (0.2-0.6); PLM-ICD maintains rank 1 at all thresholds, with minor swaps between Convolutional Attention for Multi-Label Classification (CAML) and MultiResCNN at extreme thresholds. Panel (b) shows model rank stability across false positive penalty weights (α from 0.0 to 1.0); rankings are perfectly preserved (Spearman ρ=1.0 at all α values). Rankings were also identical across DRG weighting schemes (uniform, mean, admission-specific) and RSI formulations (raw vs ratio; not shown). CRS: Coding Reimbursement Score.

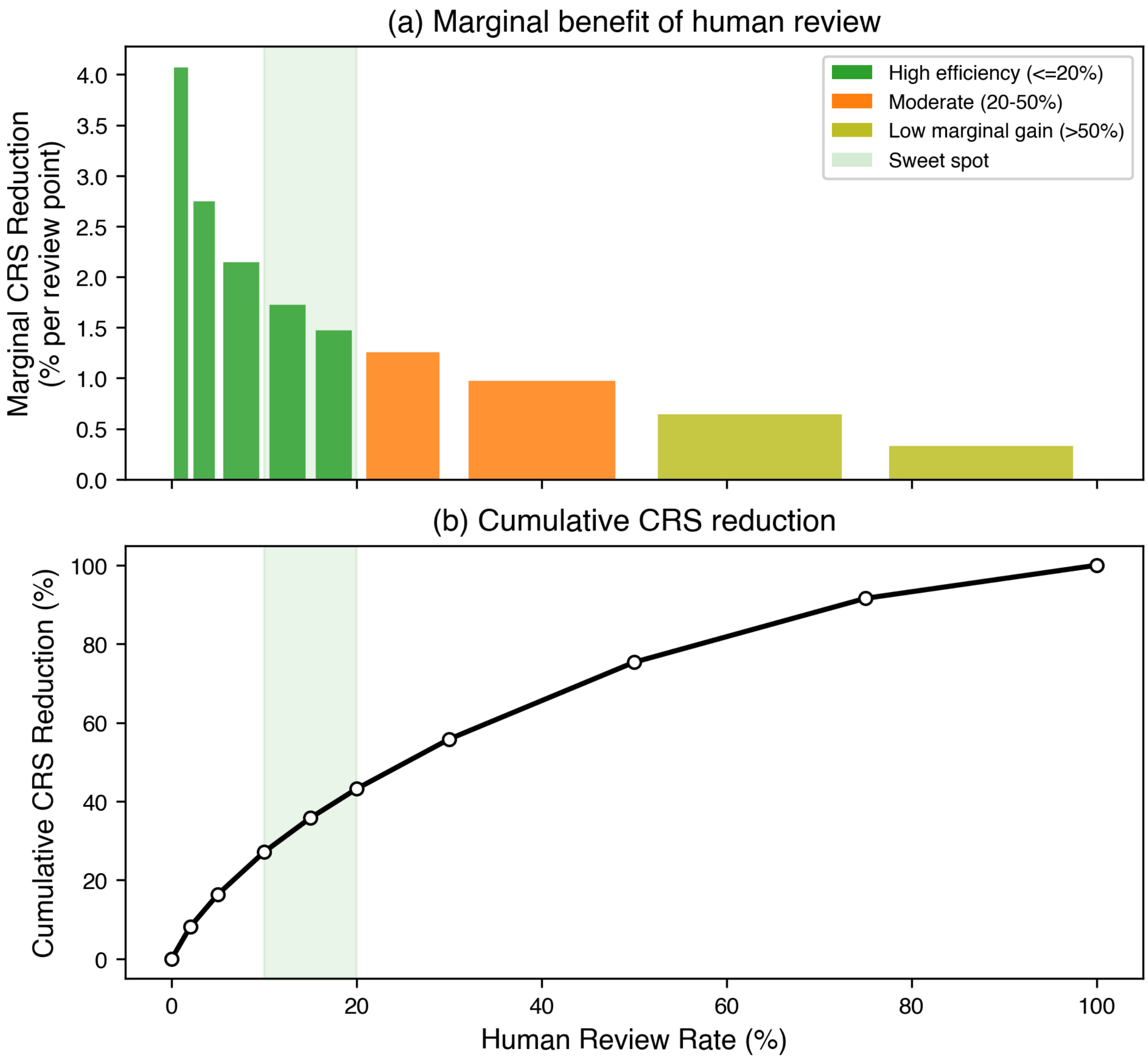

Figure 4: **Supplementary Figure S4.** Cost-effectiveness of revenue-targeted human review (S3 strategy). Panel (a) shows marginal Coding Reimbursement Score (CRS) reduction per review-rate point; green bars indicate high-efficiency segments (20% or less review rate), orange indicates moderate returns, and gray indicates diminishing marginal benefit. Panel (b) shows cumulative CRS reduction as a function of review rate. CRS: Coding Reimbursement Score.

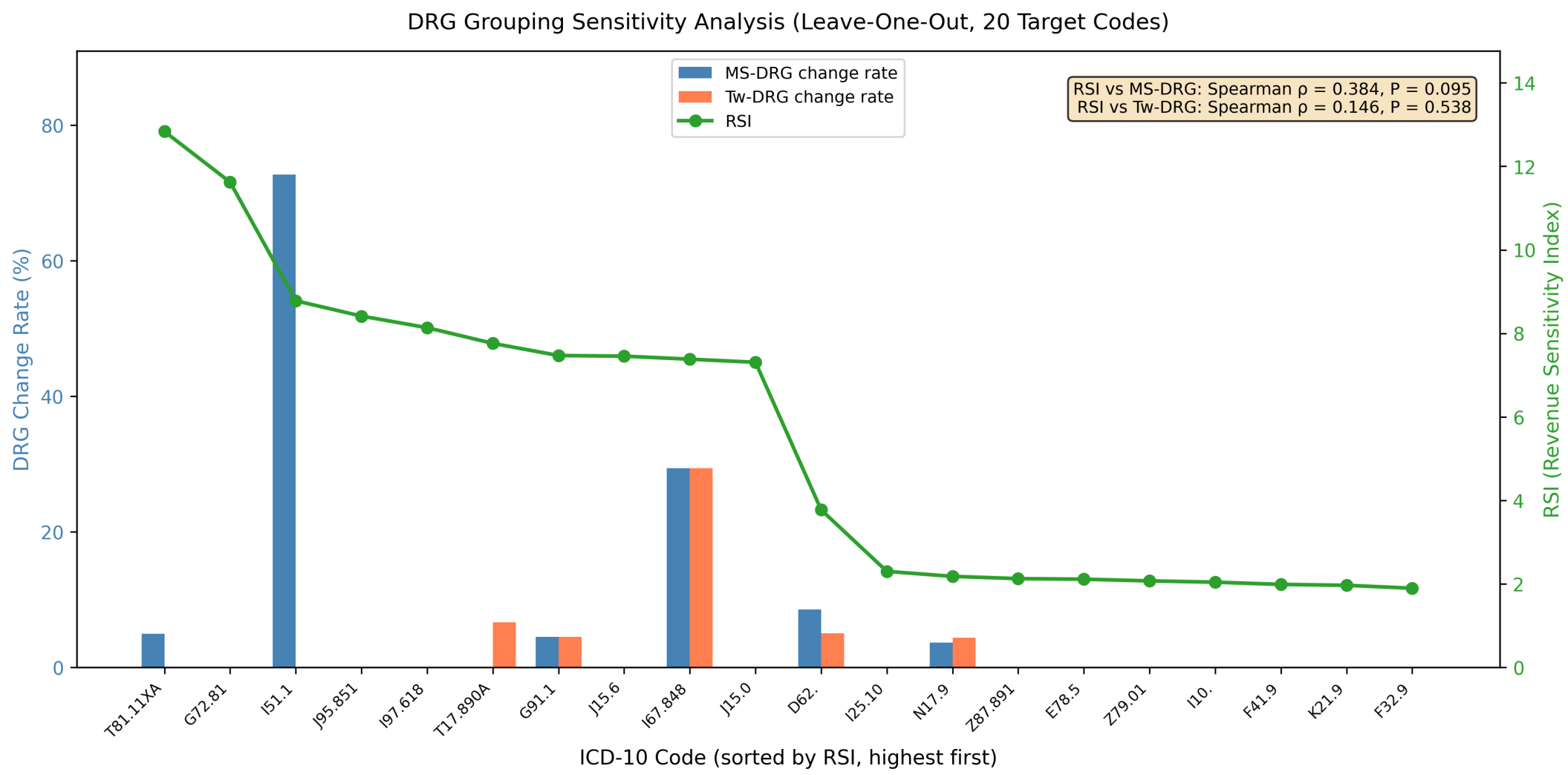

Figure 5: **Supplementary Figure S5.** DRG grouping sensitivity analysis (leave-one-out, 20 target codes sorted by RSI). Bars show the DRG change rate (%) under MS-DRG (blue) and Tw-DRG (orange) when each code is removed as a secondary diagnosis; the green line shows the corresponding RSI value (right axis). Most high-RSI codes (left side) produce zero DRG changes upon removal, confirming that RSI captures co-occurrence with high-weight admissions rather than direct causal influence on DRG assignment. Rank correlations between RSI and DRG change rate were weak and non-significant under both systems (MS-DRG: Spearman ρ=0.384, P=0.095; Tw-DRG: ρ=0.146, P=0.538).

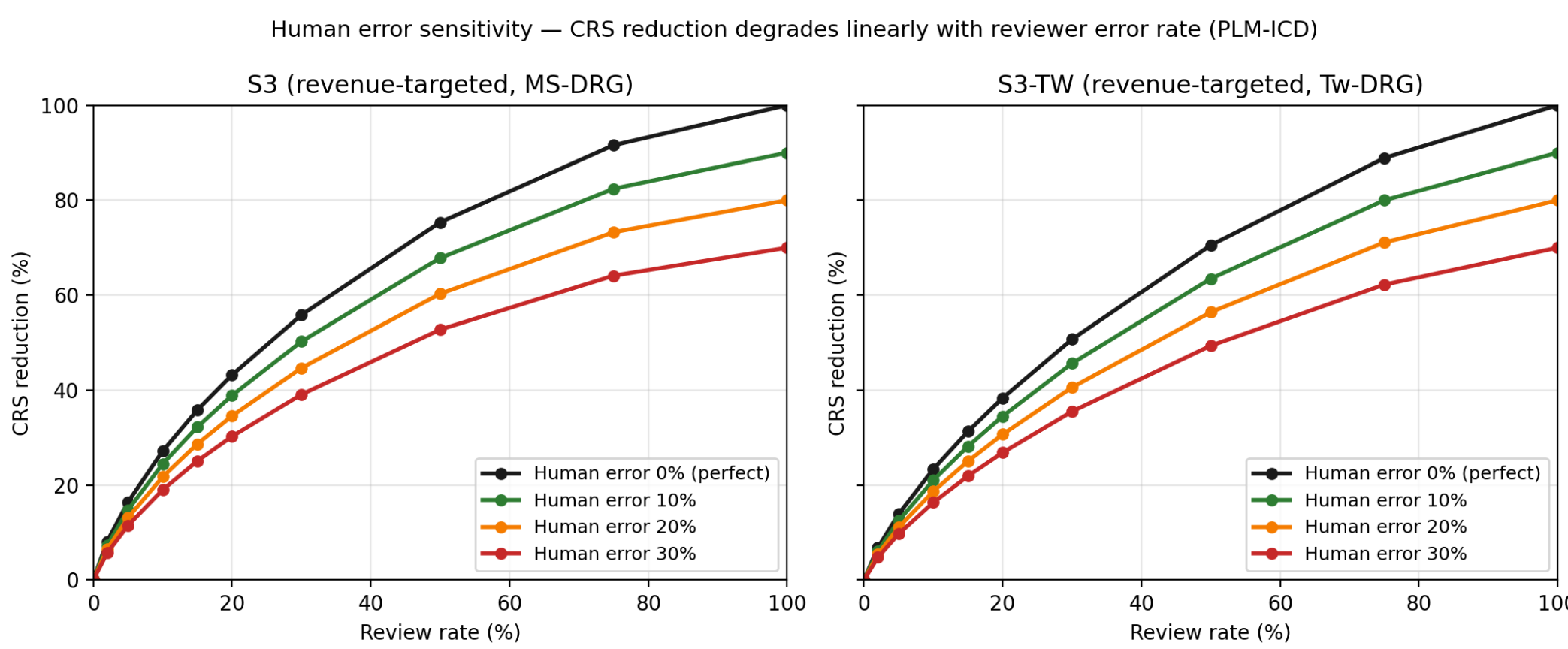

Figure 6: **Supplementary Figure S6.** Human error sensitivity analysis for revenue-targeted review (PLM-ICD). Each panel shows Coding Reimbursement Score (CRS) reduction as a function of human review rate (0%-100%), with separate lines for reviewer error rates *e* of 0% (perfect), 10%, 20%, and 30%. Left panel: S3 strategy evaluated under MS-DRG. Right panel: S3-TW strategy evaluated under Tw-DRG. Each AI error in a reviewed admission is corrected with probability $1 - e$ independently; recovered counts and CRS therefore scale linearly with $1 - e$ in expectation. At the 20% review rate, S3 achieves 43.2%, 38.9%, 34.6%, and 30.2% MS-DRG CRS reduction for e = 0%, 10%, 20%, 30% respectively. Even at the highest reviewer error rate tested (30%), revenue-targeted review retains a 1.5-fold advantage over random sampling at perfect correction (20.0%). CRS: Coding Reimbursement Score.

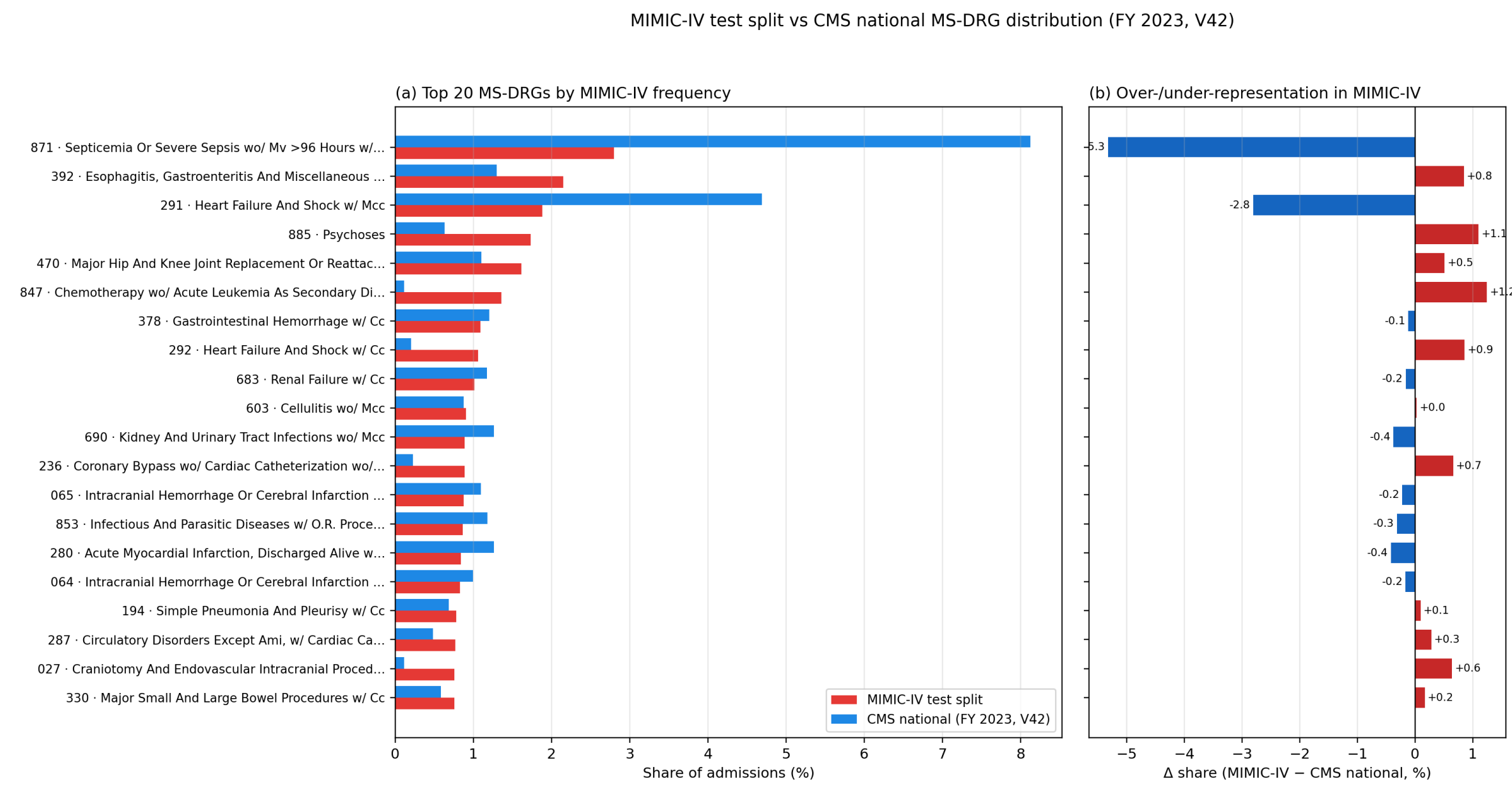

Figure 7: **Supplementary Figure S7.** MS-DRG case-mix distribution: MIMIC-IV test split (17,225 admissions with HCFA DRG assignment) versus CMS national Medicare claims (FY 2023, V42 grouper; 6,871,447 claims used for FY 2025 relative-weight calibration). **Panel (a)**: horizontal grouped bars showing, for the top 20 MS-DRGs by MIMIC-IV frequency, the share of admissions in each source (red = MIMIC-IV, blue = CMS national); rows are ordered by MIMIC-IV frequency with DRG 871 at the top. **Panel (b)**: horizontal Δ bars, same row order as Panel (a), showing the per-DRG difference MIMIC-IV − CMS national (%); red indicates over-representation and blue under-representation in MIMIC-IV, with numeric values annotated. The two distributions are strongly correlated (Spearman ρ = 0.846, $P < 10^{\square 1 \square\square}$, full-distribution rank correlation across 656 shared DRGs), but the top-20 share is more concentrated nationally (36.2%) than in MIMIC-IV (23.9%), consistent with a longer tail of specialty and high-complexity conditions at a tertiary academic centre. Notable over-representations in MIMIC-IV include Chemotherapy (DRG 847, ~12-fold), Heart Failure and Shock with CC (DRG 292, ~5-fold), and Coronary Bypass without Cardiac Catheterisation (DRG 236, ~4-fold), whereas Septicaemia or Severe Sepsis with mechanical ventilation >96 hours (DRG 871) is under-represented relative to the Medicare national case mix because the Medicare comparator covers an older and more disability-enriched population than MIMIC-IV's adult all-payer sample. These differences underscore that MIMIC-IV's case-mix is not a random draw from U.S. hospital admissions and that cross-hospital generalisation requires prospective validation. CC: complication or comorbidity.

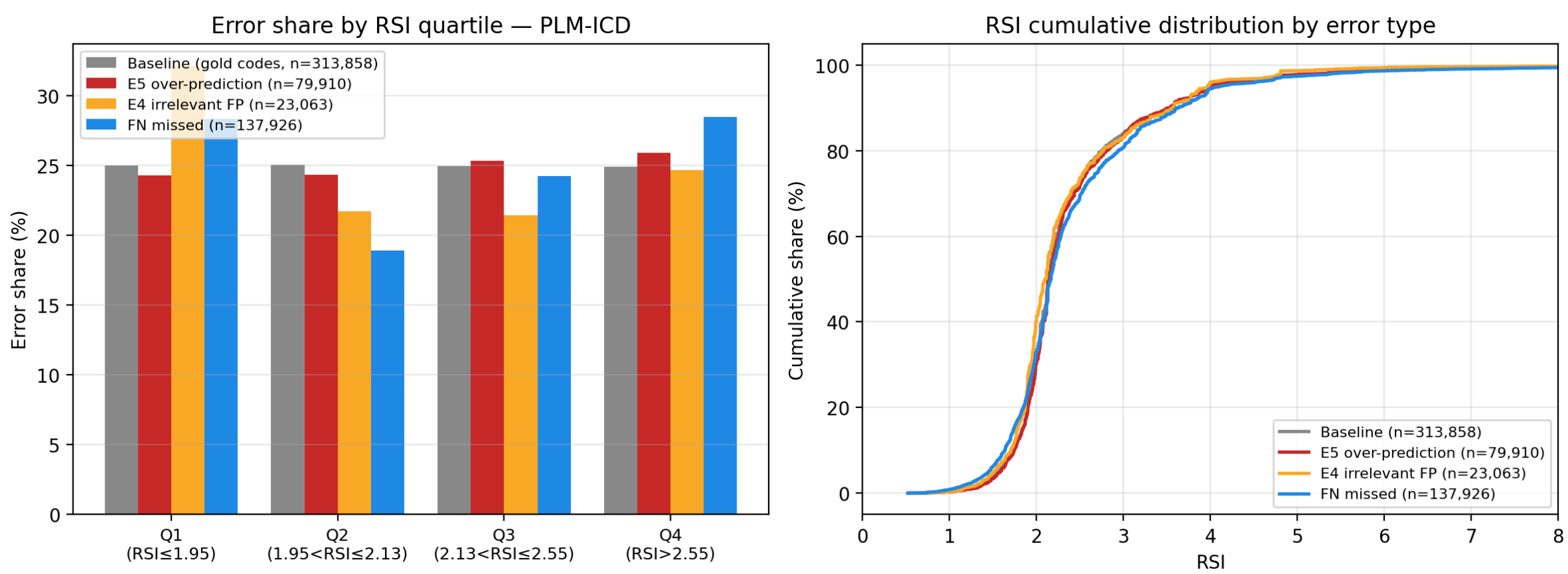

Figure 8: **Supplementary Figure S8.** E5 over-prediction × MS-DRG Revenue Sensitivity Index (RSI) cross-tabulation for PLM-ICD. Panel (a): share of errors falling in each RSI quartile of the gold-code distribution, grouped by error type — baseline (all gold code instances, n=313,858), E5 over-prediction (n=79,910), E4 irrelevant false positive (n=23,063), and false-negative misses (E1+E2+E3, n=137,926). Panel (b): cumulative RSI distribution for the same four error types. E5 errors distribute nearly uniformly across quartiles (24.3%, 24.4%, 25.4%, 25.9%) with a mean RSI (2.44) essentially identical to the baseline gold-code mean (2.42), indicating that over-prediction is not preferentially concentrated on high-RSI codes. E4 irrelevant errors are skewed toward low RSI (Q1 32.1%). The slight upper-quartile excess of E5 (25.9% vs 25.0% expected) nonetheless corresponds to approximately 20,700 over-prediction instances in the highest RSI quartile, so audit-exposure risk scales with the total volume of excess codes rather than with a differential bias toward financially impactful codes. RSI: Revenue Sensitivity Index; Q1–Q4: quartiles of the RSI distribution weighted by gold-code incidence (Q1 lowest, Q4 highest).
